# An association study of ADSS gene polymorphisms with schizophrenia

**DOI:** 10.1186/1744-9081-4-39

**Published:** 2008-08-24

**Authors:** Fuquan Zhang, Pak C Sham, Hua Fan, Yong Xu, Xuezhu Huang, Honcheong So, Yuqing Song, Pozi Liu

**Affiliations:** 1 Institute of Neurological disorders, Tsinghua University; Department of Psychiatry, Yuquan Hospital, Tsinghua University, Bejing, 100049, PR China; 2 Department of Psychiatry, University of Hong Kong, Hong Kong, PR China; 3 Department of Psychiatry, Beijing Anding Hospital, Capital Medical University, Bejing, PR China

## Abstract

**Background:**

Adenylosuccinate synthase (ADSS) catalyzes the first committed step of AMP synthesis. It was suggested that the blood-derived RNA of ADSS was down-regulated in schizophrenia (SZ) and one of the eight putative biomarker genes to discriminate SZ from normal controls. However, it remains unclear whether the reduction of ADSS RNA is due to the polymorphisms of the gene or not.

**Methods:**

We attempted to examine the association of ADSS gene with schizophrenia in a Chinese population of 480 schizophrenics and 502 normal controls. Genotyping was performed by the Sequenom platform.

**Results:**

The 6 marker SNPs (rs3102460, rs3127459, rs3127460, rs3127465, rs3006001, and rs3003211) were genotyped. The frequencies of alleles, genotypes, and haplotypes were tested between cases and controls. There was no significant difference of genotypic, allelic, or haplotypic distributions of the 6 SNPs between the two groups.

**Conclusion:**

Our data did not support ADSS gene as a susceptibility gene for SZ in Chinese Han population. Large sample size study is needed to validate or replicate our association study, especially from other ethnic populations.

## Background

Schizophrenia is a complex genetic disorder characterized by profound disturbances of cognition, emotion and social functioning. The median lifetime prevalence of SZ is 0.7%–0.8% [1]. The public health importance of SZ is clear. Numerous family, twin, and adoption studies showed conclusively that the risk of schizophrenia was increased among the relatives of affected individuals and that it was the result largely of genes rather than shared environment [2]. It's believed that the disease tends to run in families, with an estimated heritability of 80–85% [3]. In the children and siblings of individuals with schizophrenia, the increase in risk is approximately 10-fold. Recent decades, many candidate genes have been implicated in the susceptibility of SZ with independent replicative evidences from multiple populations [2,4,5].

Following the searching of genetic basis for the mechanism of SZ, some lines of evidences have also emerged from other kinds of biomarkers, such as level of gene expression. Recent advances have facilitated the use of circulating blood to conduct genomic analyses of human disease [6,7]. Vawter et al. [8] identified nine genes that were differentially expressed between SZ patients and controls. Thereafter, by analyzing the blood-derived RNA from 74 samples, linear and non-linear combinations of eight putative biomarker genes (APOBEC3B, ADSS, ATM, CLC, CTBP1, DIDO1, CXCL1, and S100A9) were able to discriminate among schizophrenia, bipolar disorder, and control samples [9], with an overall accuracy of 95%–97%. None of these genes have yet been investigated for their association or linkage disequilibrium with SZ.

Among them, ADSS is down-regulated in the SZ patients. The de novo biosynthesis of AMP from IMP involves two steps, with the first step catalyzed by ADSS followed by adenylosuccinate lyase catalyzing the second step [10]. The gene encoding ADSS maps to 1cen-q12, the chromosomal loci previously linked to schizophrenia in meta-analysis [11,12]. The sequence of ADSS gene is 44 kb in length, with 13 extrons and 12 introns.

However, it is unknown whether the alteration in ADSS expression is due to defect of the gene, or secondary to other disease-related factors. In order to address this issue, and to test the hypothesis that sequence variations of ADSS gene influence the risk for the disease, we conducted a case-control association study on 6 SNPs (rs3102460, rs3127459, rs3127460, rs3127465, rs3006001, and rs3003211) within the gene in a Chinese Han Population.

## Methods

### Subjects

Subjects were 480 unrelated schizophrenics and 502 healthy controls. Cases (age: 41.8 ± 10.3) were recruited from Hong Kong hospitals. All patients were interviewed using the Structured Clinical Interview for DSM-IV (SCID) and met the DSM-IV diagnostic criteria for schizophrenia. Healthy controls (age: 41.9 ± 9.79) were recruited from blood donors who were not screened for psychiatric diseases. However, in Hong Kong an individual would be ineligible for blood donation if he is under doctor's care, taking medications, awaiting test results or suffering from any serious illness. All subjects were Han Chinese. Peripheral blood sample were obtained from the subjects. All participants provided written informed consent.

### Genotyping

In view of information from dbSNP [13], according to the location and the heterozygosity of SNP, we selected 6 SNPs from intron-11 (rs3102460), intron-6 (rs3127459), intron-4 (rs3127460), intron-1 (rs3127465, rs3006001 and rs3003211) to check the allelic and haplotypic association of ADSS with SZ. The average estimated heterozygosity of these SNPs is 0.37, and they span 36 Kb nucleotides in the 44 Kb pairs of ADSS.

We employed a Sequenom platform (Sequenom MassARRAY system, Sequenom, San Diego CA) for assay design and genotyping. SNP sites were amplified by PCR in multiplex format in 384-microtiter plates by a pair of specifically designed forward and reverse PCR primers. The length of the amplicons for SNP capture ranged from 60 to 120 base pairs (bp). Following genomic amplification of the target regions, PCR products were treated with shrimp alkaline phosphatase for 20 minutes at 37°C to dephosphorylate any residual nucleotides and to prevent their future incorporation and interference with the primer extension assay. Extension primers, DNA polymerase, and a cocktail mixture of deoxynucleotides (dNTPs) and dideoxynucleotide triphosphates (ddNTPs) were added to each mix. These were then followed by cycles of homogeneous MassEXTEND™ (hME) reaction probed by the extension primers for each SNP. The MassARRAY™ typer software version 3.1 was then used to read out the extended mass and assign the genotype call. Quality control criteria included genotype call rate of >80%, less than 1 duplicate errors (5 duplicates in each 96 well-plate), and significant Hardy-Weinberg disequilibrium.

### Statistical analyses

Hardy-Weinberg equilibrium, genotype and allele frequencies between cases and controls for ADSS markers were tested using PLINK [14]. Linkage disequilibrium (LD) between markers was tested by Haploview [15]. Haplotype analyses were performed using UNPHASED [16] as well as SHEsis [17]. UNPHASED contains a suite of programs for association analysis of haplotype data, including COCAPHASE and QTPHASE. The most significant p-value in the haplotype analysis was corrected for multiple testing by running 1000 permutations of the data by COCAPHASE. In each permutation, the 'case' and 'control' labels were randomly re-assigned among subjects and the best p value were stored to provide an empirical frequency distribution. The program employs the expectation-maximization (EM) algorithm to estimate haplotypes with unknown phases. Individuals with missing genotype information at one or more loci were excluded from analyses by UNPHASED. Haplotypes with frequencies <3% in the whole sample were considered be to be rare and excluded.

## Results

### Genotype and allele distributions of SNPs

The allele  and genotype frequencies of 6 SNPs among 480 SZ patients and 502 healthy controls were shown in table 1. The genotypic distributions of these six polymorphisms did not deviate significantly from Hardy-Weinberg Equilibrium in both patients and controls (p > 0.05). There was no significant difference in genotype or allele frequencies between cases and controls.

**Table 1:** Genotypic and allelic distributions of the ADSS SNPs in cases and controls

SNP		Genotype frequency (%)		p (df = 2)		Allele frequency (%)		p(df = 1)	OR(95%CI)	HW p
		CC	CT	TT		C	T			
										
Rs3102460	ca441	48(10.9)	176(39.9)	217(49.2)	0.442	272(30.8)	610(69.2)	0.344	1.1(0.9–1.35)	0.181
	co443	37(8.4)	181(40.9)	225(50.8)		255(28.8)	631(71.2)			1
										
		AA	AT	TT		A	T			
										
Rs3127459	ca476	26(5.5)	170(35.7)	280(58.8)	0.425	222(23.3)	730(76.7)	0.212	0.88(0.71–1.08)	1
	co499	36(7.2)	185(37.1)	278(55.7)		257(25.8)	741(74.2)			0.484
										
		TT	AT	AA		T	A			
										
rs3127460	ca477	12(2.5)	149(31.2)	316(66.2)	0.740	173(18.1)	781(81.9)	0.487	0.92(0.74–1.16)	0.353
	co501	16(3.2)	162(32.3)	323(64.5)		194(19.4)	808(80.6)			0.477
										
		CC	CT	TT		C	T			
										
rs3127465	ca470	11(2.3)	144(30.6)	315(67.0)	0.673	166(17.7)	774(82.3)	0.551	0.93(0.74–1.18)	0.340
	co489	16(3.3)	151(30.9)	322(65.8)		183(18.7)	795(81.3)			0.882
										
		CC	AC	AA		C	A			
										
rs3006001	ca475	12(2.5)	146(30.7)	317(66.7)	0.680	170(17.9)	780(82.1)	0.409	0.91(0.72–1.14)	0.434
	co496	16(3.2)	160(32.3)	320(64.5)		192(19.4)	800(80.6)			0.565
										
		GG	AG	AA		G	A			
										
rs3003211	ca436	50(11.5)	171(39.2)	215(49.3)	0.668	271(31.1)	601(68.9)	0.401	1.09(0.89–1.34)	0.093
	co453	44(9.7)	177(39.1)	232(51.2)		265(29.2)	641(70.8)			0.256

ca = case, co = control. HW = Hardy-Weinberg

### Patterns of LD

**Figure 1:**
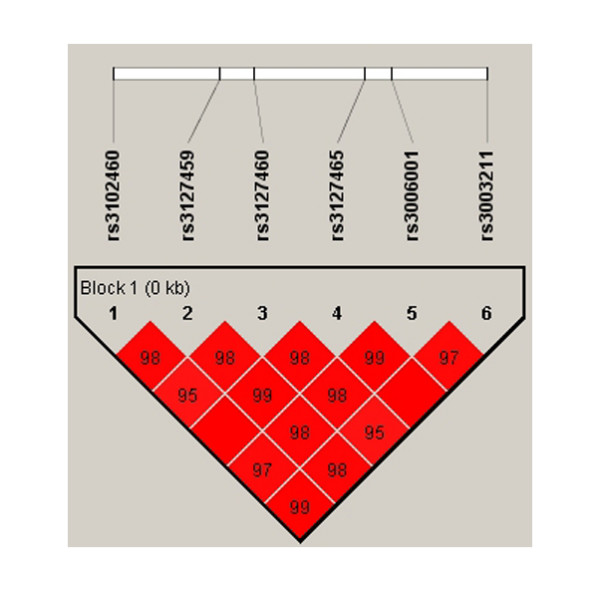
**Graphical representation of LD patterns of the six SNPs in the total sample**. The graphic represents the pairwise calculation of r^2 ^for each pair of SNPs at each gene. Squares represent r^2 ^values. Values within boxes are the measures of r^2 ^between pairs of markers in a scale from 0 to 100. If there is no value, r^2 ^= 1.

The patterns of Pair-wise LD between neighbouring SNPs were shown in figure 1. Fairly tight linkage disequilibrium was observed in any pair of the SNPs and the six markers were in strong LD.

### Haplotypes of the six markers

As can be seen in table 2, there is no global or individual significant difference for 6-marker haplotype in our sample. Individual haplotype tests were performed by evaluating the difference in risk between a specific haplotype and all others grouped together. Also, we obtained no significant difference in haplotype frequencies for two-, three-, four-, or five-marker analyses between patients and controls (data not shown).

**Table 2:** Estimated 6-SNPs haplotype frequencies and association significance

Haplotype	Case(freq)	Control(freq)	χ^2^	p	OR (95%CI)	Global p
TTATAA	372(46.2)	364.7(45.0)	0.119	0.731	1.035 (0.850–1.260)	0.339
CTATAG	243(30.1)	221.3(27.3)	1.388	0.239	1.139 (0.917–1.413)	
TATCCA	140(17.4)	156(19.3)	1.087	0.297	0.874 (0.679–1.126)	
TAATAA	45(5.6)	57(7.0)	1.523	0.217	0.776 (0.518–1.162)	

Freq = frequency. Haplotypes were omitted if the estimated haplotype probability was less than 3%.

## Discussion

Although genome-wide scan for linkage in multiply-affected families with schizophrenia has consumed lots of money and effort, no forms of schizophrenia following Mendelian inheritance patterns have yet been discovered [2]. Numerous evidences show that multiple genes of small effect contribute to increasing liability to SZ, which seems to have a cumulative effect, and putatively, threshold effect. On the other hand, the study on schizophrenia has confirmed the vital role of genes in its etiology, but has not so far identified the relationship between observed genetic risks and specific DNA variants, protein alterations, or biological processes [18], nor was diagnostic neuropathology, or even unequivocally replicated associations with the same alleles or haplotype within each gene identified [4].

Meta-analyses of SZ linkage studies have provided evidence of susceptibility in chromosomes 2p, 5q, 3p, 11q, 6p, 1q, 22q, 8p, 20q, 22q, and 14p [2,12], some of which are strong enough to withstand rigorous correction for whole genome analysis (e.g. 1q, 6p, 6q, 15q) [19]. Recently, many polymorphisms located in regions of chromosome 1 were reported as susceptibility loci for SZ [5,20], including risk markers in 1q44 [21] 1q43 [22], both of which lie adjacent to ADSS.

Although whole genome linkage scan and functional studies have implied several very promising positional candidates, such as NRG1 [23], DTNBP1 [24], COMT [4], DISC1 [25], DAOA [26,27], RGS4 [28] and so on [5,23,28-31], there may be several genes that play subtle or weak roles in the pathogenesis of SZ, making it difficult to investigate by traditional approaches. To tackle this problem, another approach to finding potential candidate genes is using microarray technology to examine RNA of a gene which shows differential expression between patients and controls, by which ADSS gene was suggested as one of candidates for SZ.

Functionally, the ADSS catalyzes the key step in the synthesis of AMP, which influences the energy metabolism through the purine nucleotide cycle (PNC) and the AMPK (AMP-activated protein kinase) pathway [32]. The reaction is as follows: IMP+L-aspartate+GTP ↔ adenylosuccinate+GDP+phosphate. Genetically, ADSS gene resides in the susceptibility loci and the blood-derived RNA of ADSS was among the eight genes with putatively differential diagnostic power for SZ. Hereto, there is no genetic association study of ADSS with SZ, and the mechanism of hypo-expression of ADSS is elusive, nevertheless, it suggested that ADSS gene was possibly involved in the genetic architecture of SZ, for genetic variation or polymorphisms can dominate gene expression as well as confer disease risk to human beings.

Our study is an exploration study to seek the trait's genetic basis following a clue from RNA alterations in SZ. However, the data did not display any statistically significant difference for both the allelic and genotypic distribution between cases and controls. Although haplotype analysis is a powerful tool for identifying candidate genes for complex disease, there was no positive finding in current study. Therefore, our result does not support the association of ADSS gene with SZ. It implies that down-regulation of ADSS in patients may not result from the gene's polymorphisms.

One common issue in the study of complex diseases is the limited sample size, resulting in inadequate power to detect association. With regard to our data, assuming the frequency of risk allele in controls to be 0.5, our sample of 480 cases and 502 controls is able to detect an odds ratio (OR) of 1.43 or above with 80% power [33]. It is possible that false-negative results may arise from lack of power. Another issue of researches in multifactor diseases is the underlying high degree of genetic heterogeneity of perplexing phenotype. SZ is not simply defined by several major genes but rather evolves from addition or potentiation of a specific cluster of genes, which subsequently determine the genetic vulnerability of an individual. Thus, there are likely different genes or sets of genes associated with schizophrenia disorders in different populations [34]. It is necessary to validate or replicate our association results using independent samples especially from other ethnic populations.

It's been suggested that the promoter and 5' flanking region of murine ADSS gene possessed regulatory elements that could influence its expression [35]. In addition, ADSS has several splicing variants. Therefore, the examinations of SNPs within 5' flanking region and those potentially affecting splicing may be valuable in further study.

## Conclusion

In conclusion, our data did not support ADSS gene as a susceptibility gene for SZ in Chinese Han population. Further study on genetic regulation, neural network, or epigenetics will facilitate to elucidate the role of ADSS in SZ.

## Abbreviations

APOBEC3B: apolipoprotein B mRNA editing enzyme, catalytic polypeptide-like 3B; ATM: ataxia telangiectasia mutated; CLC: Charcot-Leyden crystal protein; COMT: catechol-O-methyltransferase; CTBP1: C-terminal binding protein 1; CXCL1: chemokine (C-X-C motif) ligand 1; DAOA: D-amino acid oxidase activator; DIDO1: death inducer-obliterator 1; DISC1: disrupted in schizophrenia 1; DTNBP1: dystrobrevin binding protein 1; NRG1: neuregulin 1; RGS4: regulator of G-protein signaling 4; S100A9: S100 calcium binding protein A9.

## Competing interests

The authors declare that they have no competing interests.

## Authors' contributions

SPC designed and coordinated the study. ZFQ, XY, and SYQ drafted the manuscript. FH, SHC and HXZ participate in data management and statistical analysis. LPZ participated in the supervision of the project. All authors read and approved the final manuscript.
